# Supplementary material for: Sex-specific associations between person and environment-related childhood adverse events and levels of cortisol and DHEA in adolescence
Source: PLoS One. 2020 Jun 4;15(6):e0233718. doi: 10.1371/journal.pone.0233718 (PMC7272021; doi:10.1371/journal.pone.0233718)
Supplement: Attachment — Submitted filename: Response to reviewer 2020Mar24.docx [file pone.0233718.s002.docx]

Dear Dr. Alexandra Kavushansky,

We would like to thank the editor and reviewer for their time and the useful comments regarding the manuscript ‘**Sex-specific associations between person and environment-related childhood adverse events and levels of cortisol and DHEA in adolescence**’. Please find our answers to the comments and questions below.

**Editor comments:**

1. How LC-MS/MS and ELIZA analyses were performed?

*We have clarified the methods in the methods section. The LC-MS/MS assays were performed based on the method as described in the following paper: Gao, W., Stalder, T., Foley, P., Rauh, M., Deng, H., & Kirschbaum, C. (2013). Quantitative analysis of steroid hormones in human hair using a column-switching LC–APCI–MS/MS assay. Journal of Chromatography B., 928, 1–8. doi:10.1016/j.jchromb.2013.03.008*

*ELIZA: Analysis of cortisol concentrations was carried out by means of salivary cortisol immunoassay with time-resolved fluorescence endpoint detection. (as described in Dressendorfer et al., 1992)*

*All analyses were performed by Dresden Labservices in Germany.*

2. How the normality of the data distribution was tested?

*Normality was assessed by visual inspection of histograms and Kolmogorov-Smirnov tests. We have added this information in the statistical analyses section.*

3. Was the person performing the analyses blind to the experiment (e.g. participants` groups) details?

*No, the person performing the analyses was not blind to the groups since the groups were not defined before the study started but based on outcomes of the study and by the same person who performed the analyses. Groups were defined before analysis of the lab outcomes though, so we are sure that this has not affected the study outcomes.*

4. How was the participants` conformity to following the instructions for gathering and preserving the saliva samples verified? How were the samples delivered to the lab? (frozen?)

*Participants received detailed instructions, in both text and pictures, about the collection of the saliva samples. They were asked to provide the actual time of collecting the sample, also if the time deviated from the time they were supposed to collect the sample. However, we cannot be sure if they truly did so. Conformity to the instructions was not specifically verified.*

*Samples were sent unfrozen by mail and were immediately stored in the freezer until the assays were performed. We have added this information to the methods section.*

5. Why only half of the initial subjects` sample was tested for the saliva measures?

*Only half of the initial group was invited to participate in the second part of the study; the saliva sample part. There was not enough funding available to assay saliva samples for the entire group.*

6. There are several language errors, such as: "…significant lower levels (line 234)", " boys compared than girls (line 270)", etc.

*We thank the editor for spotting these mistakes and changed it to “significantly lower levels” and “boys compared to girls”. We have also thoroughly read the manuscript again looking for other language errors but think there are no other errors.*

7. Graphs do not show neither significant results, nor trends.

*We have included the P values in the graphs.*

**Journal requirements:**

1. Please ensure that your manuscript meets PLOS ONE's style requirements, including those for file naming. The PLOS ONE style templates can be found at http://www.plosone.org/attachments/PLOSOne_formatting_sample_main_body.pdf and <http://www.plosone.org/attachments/PLOSOne_formatting_sample_title_authors_affiliations.pdf>

*We have attached the figures separately.*

2. We note that you have stated that you will provide repository information for your data at acceptance. Should your manuscript be accepted for publication, we will hold it until you provide the relevant accession numbers or DOIs necessary to access your data. If you wish to make changes to your Data Availability statement, please describe these changes in your cover letter and we will update your Data Availability statement to reflect the information you provide.

*We have included a dataset.*

3. PLOS requires an ORCID iD for the corresponding author in Editorial Manager on papers submitted after December 6th, 2016. Please ensure that you have an ORCID iD and that it is validated in Editorial Manager. To do this, go to ‘Update my Information’ (in the upper left-hand corner of the main menu), and click on the Fetch/Validate link next to the ORCID field. This will take you to the ORCID site and allow you to create a new iD or authenticate a pre-existing iD in Editorial Manager. Please see the following video for instructions on linking an ORCID iD to your Editorial Manager account: <https://www.youtube.com/watch?v=_xcclfuvtxQ>

*We have added an ORCID ID for the corresponding author.*

4. We note that you have included the phrase “data not shown” in your manuscript. Unfortunately, this does not meet our data sharing requirements. PLOS does not permit references to inaccessible data. We require that authors provide all relevant data within the paper, Supporting Information files, or in an acceptable, public repository. Please add a citation to support this phrase or upload the data that corresponds with these findings to a stable repository (such as Figshare or Dryad) and provide and URLs, DOIs, or accession numbers that may be used to access these data. Or, if the data are not a core part of the research being presented in your study, we ask that you remove the phrase that refers to these data.

*We have removed the phrase “data not shown” and replaced it with the results.*

**Reviewer comments:**

1.Results Section

1.1 It would be helpful for the reader if the significant differences of table 2 were clearly indicated, along with the respective p value.

*We have marked the significant differences and included the P value.*

1.2 Authors use terms like similar and comparable, instead of no significant differences. Please indicate the p values to clearly indicate that comparable means not significantly different.

*We have changed the terminology to clearly indicate the level of significance.*

1.3 The term “trend” appears in the results and discussion section, but the p value is not shown in the tables or text. Please insert the p value.

*P value has been inserted.*

2 Discussion section

2.1 Please explain the sentence below in more detail in the discussion section.

“These findings collectively indicate that different types of childhood adversity may be linked to blunted daily cortisol output, but overall prolonged elevated levels of cortisol, suggesting HPA-axis dysregulation, with possible future associations with impaired mental wellbeing.”

How can a daily blunted cortisol lead to an increase in hair cortisol? Would it not be expected that lower daily cortisol levels are associated with lower hair cortisol? How can the results of previous research focusing on non-specific childhood adversity and childhood daily cortisol output be reconciled with the higher hair cortisol shown in this study?

*Previous research has shown that childhood adversity is linked to blunted diurnal cortisol levels but also to increased cortisol levels in response to a stressor. This could be an explanation for our findings of blunted daily cortisol levels and overall elevated levels of cortisol (measured in hair).*

2.2 Are the size effects found in this study for the association of DHEA with person-related childhood adversity in girls, and for the association of hair cortisol and person-related childhood adversity in boys, of clinical relevance? Please compare with the size effect of the association of DHEA and cortisol with the pubertal stage and with other studies, if possible.

*We have compared the effect size of the association between childhood adversity and DHEA and cortisol to the effect size of pubertal stage and described the results in the discussion section.*

2.3 Was the association of the hair cortisol with the AUCg evaluated? If not, please explain why. If it was evaluated, please state the results clearly in the text.

*Yes, it was evaluated, and the results are described in the descriptive analyses section in the results section (lines 217-220).*
