# Supplementary material for: Sex-specific associations between person and environment-related childhood adverse events and levels of cortisol and DHEA in adolescence
Source: PLoS One. 2020 Jun 4;15(6):e0233718. doi: 10.1371/journal.pone.0233718 (PMC7272021; doi:10.1371/journal.pone.0233718)
Supplement: Attachment — Submitted filename: Response to the reviewers round 2.docx [file pone.0233718.s003.docx]

Dear Dr. Alexandra Kavushansky,

We would like to thank the reviewer for their time and the useful comments regarding the manuscript ‘**Sex-specific associations between person and environment-related childhood adverse events and levels of cortisol and DHEA in adolescence**’. Please find our answers to the comments and questions below.

**Reviewer comments:**

2 Discussion section

2.1 Please explain the sentence below in more detail in the discussion section.

“These findings collectively indicate that different types of childhood adversity may be linked to blunted daily cortisol output, but overall prolonged elevated levels of cortisol, suggesting HPA-axis dysregulation, with possible future associations with impaired mental wellbeing.”

*We agree that this sentence needs more explanation and we have changed the discussion section regarding this subject.*

How can a daily blunted cortisol lead to an increase in hair cortisol? Would it not be expected that lower daily cortisol levels are associated with lower hair cortisol?

*We agree that it would be expected that lower daily cortisol levels are associated with lower hair cortisol levels. We found additional literature regarding the correspondence between hair and salivary cortisol levels and in these two papers no significant correlation was observed between hair cortisol levels and salivary cortisol measured on a single day (including the AUCg). Based on these papers we changed the discussion section regarding this point.*

How can the results of previous research focusing on non-specific childhood adversity and childhood daily cortisol output be reconciled with the higher hair cortisol shown in this study?

*See the discussion section lines 304-315 for our changes.*

The authors did not answer the questions bellow:

- How can a daily blunted cortisol lead to an increase in hair cortisol?

*There is a weak link between hair cortisol and cortisol measured in saliva on a single day. In order to answer this question, we need to include salivary cortisol levels measured on multiple days, but these samples were not collected, unfortunately. We have also discussed this as a limitation to our study (see Strengths and Limitations paragraph).*

- Would it not be expected that lower daily cortisol levels are associated with lower hair cortisol?

*Yes, see above.*

- How can the results of previous research focusing on non-specific childhood adversity and childhood daily cortisol output be reconciled with the higher hair cortisol shown in this study?

*See above.*

Note that the question is about low daily cortisol and higher hair cortisol.

Even if there is no conclusive explanation to these questions, the authors should discuss the apparent contradictory results in daily cortisol and hair cortisol. Are there other studies, with children or other developmental stages, that show increased hair cortisol and blunted daily cortisol? These questions are important because hair and daily cortisol represent different time windows, i.e., chronic versus acute cortisol levels. Moreover, the decrease in cortisol seen in this study didn’t reach statistical significance (p=0.08). Is the conclusion about blunted cortisol levels in this study perhaps overestimated?

*We agree that the trend level significance should be interpreted with more caution. See the discussion section for our changes in lines 310-312.*

2.3 Was the association of the hair cortisol with the AUCg evaluated? If not, please explain why. If it was evaluated, please state the results clearly in the text.

Below is the answer given by the authors:

“Yes, it was evaluated, and the results are described in the descriptive analyses section in the results section (lines 217-220).”

Please note that the question is about the association of the hair cortisol with the AUCg, whereas the answer refers to the comparison of hair cortisol samples and the separate saliva cortisol samples (not to the AUCg, as asked). The analysis of the association between AUCg and hair cortisol would perhaps be helpful to the answer of comment 2.1.

*See lines 219-220 in the results section for the AUCg correlation with hair cortisol: “hair cortisol over the last month was not associated with AUCg cortisol levels (r = 0.11, p = 0.28).”*
